# A tutorial for software development in quantitative proteomics using PSI standard formats^☆^

**DOI:** 10.1016/j.bbapap.2013.04.004

**Published:** 2014-01

**Authors:** Faviel F. Gonzalez-Galarza, Da Qi, Jun Fan, Conrad Bessant, Andrew R. Jones

**Affiliations:** a Institute of Integrative Biology, University of Liverpool, Liverpool, UK; b Bioinformatics Group, Cranfield Health, Cranfield University, Cranfield, UK

**Keywords:** Quantitative proteomics, Software, Standard formats, APIs

## Abstract

The Human Proteome Organisation — Proteomics Standards Initiative (HUPO-PSI) has been working for ten years on the development of standardised formats that facilitate data sharing and public database deposition. In this article, we review three HUPO-PSI data standards — mzML, mzIdentML and mzQuantML, which can be used to design a complete quantitative analysis pipeline in mass spectrometry (MS)-based proteomics. In this tutorial, we briefly describe the content of each data model, sufficient for bioinformaticians to devise proteomics software. We also provide guidance on the use of recently released application programming interfaces (APIs) developed in Java for each of these standards, which makes it straightforward to read and write files of any size. We have produced a set of example Java classes and a basic graphical user interface to demonstrate how to use the most important parts of the PSI standards, available from http://code.google.com/p/psi-standard-formats-tutorial. This article is part of a Special Issue entitled: Computational Proteomics in the Post-Identification Era. Guest Editors: Martin Eisenacher and Christian Stephan.

## Highlights

• A tutorial to help software developers use PSI standard formats.
• A description of programming interfaces and tools available.
• Code snippets and a basic graphical interface to assist understanding.

## 1 Introduction

**Fig. 1:**
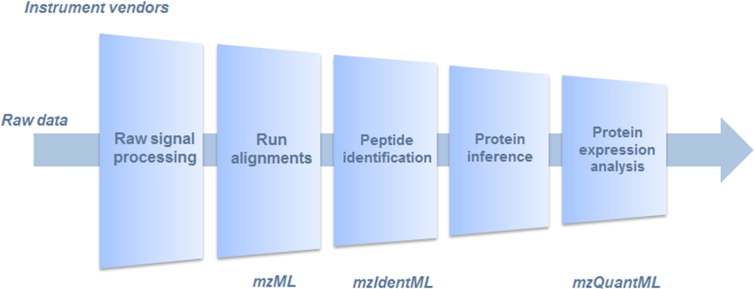
Prototypical workflow for a label free quantitative analysis, showing which stages are covered by different PSI formats.

The large scale identification and quantification of proteins in proteomics studies have always relied upon a close association with computational developments termed ‘proteome bioinformatics’ [1]. This field, also known as ‘computational proteomics’, involves the development of methods, algorithms, databases, visualisation techniques and high-throughput analysis to interpret large scale experimental studies [2–4]. This has been necessitated as mass spectrometry (MS) data, used in the identification and/or quantification of proteins, which are complex to interpret [5,6]. At present, there is no all-in-one software solution in quantitative proteomics, with huge variability in the protocols employed in different labs related to protein or peptide separation, labelling protocols and MS instruments. A full description of methods and software for protein identification/quantification is out of scope for this article, but for more details see [7–10]. The complexity in data analysis may vary considerably amongst techniques [9], for example, the following list describes the different stages that may be performed in a ‘label-free’ peptide feature-based quantification pipeline: (i) raw signal processing to locate (and potentially quantify) peptides in a two-dimensional data set, i.e. retention time (RT) versus mass/charge (*m*/*z*); (ii) alignment of parallel runs in the RT dimension; (iii) identification of a peptide sequence via a peptide-spectrum match (PSM), by querying a peptide's fragmentation pattern (MS^2^ spectrum) against a sequence database; (iv) processing a number of PSMs to infer the presence of proteins; (v) statistical analysis of differential expression and so on (Fig. 1). This example serves to illustrate the diverse types of analysis that may be performed to obtain protein expression values (i.e. quantified proteins in one or more samples) from raw MS data. Current research is focussed on the optimisation of all of these stages by the vendors of instruments, vendors of commercial analysis software or by proteome bioinformatics research groups [11–14].

The Human Proteome Organisation — Proteomics Standards Initiative (HUPO-PSI, or simply PSI) is a consortium of academic and industrial research groups, instrument manufacturers, commercial software vendors and other stakeholders aiming to standardise how proteomics data sets are reported and shared [15]. The need for the PSI's efforts came about since, historically, proteome bioinformatics has been impeded by the diverse range of data formats used for representing raw data (e.g. instrument vendors' proprietary formats or open-source formats), partially processed data for peptide identification (i.e. peak lists in text-based formats — e.g. MGF, dta, pkl, etc.), results of search engines and results of quantification software (see review in [12]). The most well-known open formats developed in the past include mzXML (for raw spectral data), pepXML (peptide identifications) and protXML (for protein identifications), developed as part of the Trans-Proteomic Pipeline (TPP) [16]. The developers of these formats have joined the PSI to develop a suite of international standards, developed in a collaborative manner.

In the context of the PSI, several high-profile outputs have been made. In the experimental proteomics domain, these include guidelines describing the minimum information that should be reported about a proteomics experiment — the parent ‘MIAPE’ document [17] and a set of technology specific modules [18–23]. The PSI has also produced standard data formats — mzML for raw or processed MS data [24], TraML for input transitions in selected reaction monitoring (SRM) approaches [25], mzIdentML for peptide and protein identification data [26] and two new efforts for capturing quantitation data — mzQuantML capturing a detailed trace of each stage of quantitative analysis [27] and mzTab capturing a simple summary of final results designed for viewing in spreadsheets or statistical processing software [28]. All of the standard formats have been designed to allow the capture of MIAPE-compliant details according to the corresponding module, but typically the formats can also be valid in different contexts if they contain more or less detail than stipulated by MIAPE documents.

With the exception of mzTab, the PSI standards are represented in Extensible Markup Language (XML) — an industry standard specification in which data are enclosed in opening and closing brackets (called elements or tags) that describe the type of data stored e.g. < spectrum > *the spectral data*</spectrum >. In XML documents, nesting of elements is allowed to build up a hierarchical tree structure so that complex concepts can be represented in a manner that can be interpreted by other developers and by software. XML files have several advantages over other formats such as platform independency, no limit on the number/type of tags defined, and that they can be easily manipulated with a large number of free software tools helping in the design, processing and visualisation of data. XML files are also known to have some disadvantages including files being relatively verbose compared with other encodings, and they tend to require specialist software to be developed for data manipulation and visualisation. Despite these constraints, XML is generally preferred by the PSI because software can be developed using industry standard tools and the format can be formally defined via an XML Schema.

All of the standards described here make use of a common controlled vocabulary (CV), called the PSI-MS CV [29], containing more than 2000 well-defined terms describing all aspects of proteomics analysis — instruments and their parts, software and parameters, etc. CV terms are used within the format to ensure that concepts can be described using standardised terminology, comprehensible to both people and software, and additional validation software has been implemented by the PSI to verify that CV terms are used correctly within formats [30].

The requirement to work with large files (> 10 GB) and fast parsing has lead bioinformatics groups to work on applications to handle these tasks. For each PSI format, various implementations have been developed for import/export from commercial and open-source software, plus software interfaces (Application Programming Interfaces or APIs) to assist developers to implement standards in their own analysis software. In this domain, the ProteoWizard project [31] has produced several software utilities for converting most proprietary vendor formats into mzML and some search engine output formats into mzIdentML. ProteoWizard also contains an internal data model (in C++) for working with MS or identification data, allowing developers to create tools without requiring an underlying knowledge of the source data format. Various groups have also collaborated to build APIs in Java for processing each of the standards, called jmzML [32], jTraML [33], jmzIdentML [34], jmzQuantML [35] and jmzTab [36]. The Java APIs have read/write capabilities and implement random-access strategies allowing files of any size to be processed without requiring the whole file to be loaded into memory.

In this article, we briefly review the model behind three of the core (XML-based) formats — mzML, mzIdentML and mzQuantML, focusing on the most important features that developers should be aware of when implementing support for them in software. The mzTab standard is not covered in this article, since, due to its limited content and simpler tab-separated structure, it is more straightforward for developers to work with. We then illustrate how the corresponding Java APIs can be used to develop support for these formats rapidly, for a range of common tasks that may be employed in a quantitative proteomics pipeline. We anticipate that this article will serve as a useful guide to proteome bioinformatics developers as to how the formats can be easily supported and integrated into existing or new software workflows.

## 2 Data standards and programming interfaces

A variety of quantitative proteomics approaches have become increasingly popular over the last few years, with a wide range of software supporting some or all approaches (reviewed in [37]). In this section, we discuss the basic usage of three main PSI standards in a quantitative analysis pipeline and how potential users of the standards can convert their own files into the standards (Table 1). The PSI standards are all maintained in regularly updated subversion repositories on Google Code. In addition to the published articles [24,26], basic tutorial documents and full specifications are available. For more details, consult the PSI website and follow appropriate links at http://www.psidev.info/.

### 2.1 Converting MS data into mzML and accessing spectral data

Each instrument vendor exports raw data into their own file format. In some cases, these companies have developed their own tools for converting these proprietary files into readable text or XML formats; however, full export to the most recent PSI standards is not yet available for several vendors. To assist bench scientists in the conversion of different vendor formats, a number of software packages have been developed by different groups. For example ProteoWizard [31] can convert most of the common vendor files such as .RAW (Thermo Scientific), .raw (Waters), .wiff (Applied Biosystems), .d (Agilent), and others into mzML via its graphical user interface (GUI) called MSConvertGUI or using the command line mode. ProteoWizard also has options for import/export of mzXML [38] and text-based peak list formats such as Mascot Generic Format (MGF). Users can also apply several features or tools, e.g. select only MS^1^ or MS^2^ data, create a subset based on a given RT or *m*/*z* range or perform peak picking to convert raw/profile data into centroid data. This feature is particularly useful in the context of making an input file suitable for searching, where profile MS^2^ data is not usually required.

**Fig. 2:**
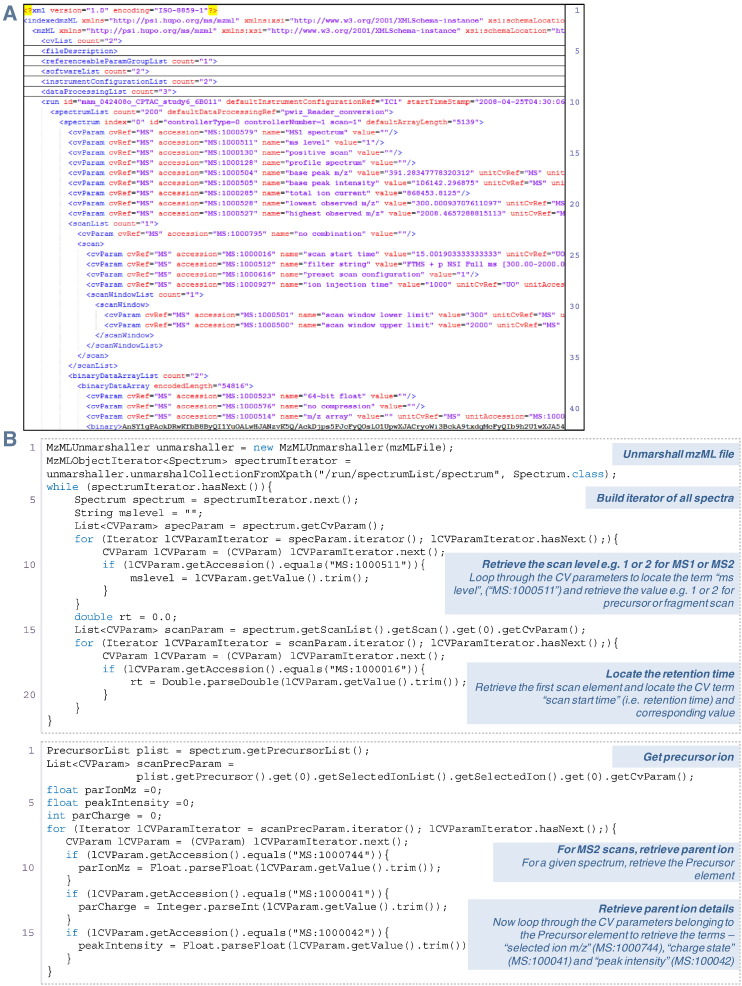
(A) A portion of an example mzML file showing file-level metadata (lines 1–9), a single spectrum (lines 12 onwards), metadata for the spectrum (lines 13–21), details of a given scan (24–34) and raw *m*/*z* data (line 42). (B) Examples of Java code snippets for using jmzML to extract particular details from an mzML raw file. Full source code available at: http://code.google.com/p/psi-standard-formats-tutorial/.

The mzML standard format is perhaps the simplest of the PSI's XML-based formats in terms of its structure. The file contains the raw data from the mass spectrometer, i.e. it comprises the *m*/*z* and intensity values for each of the scans. The schema is divided into two main sections, (i) the metadata section consisting of several headers such as the fileDescription, softwareList, instrumentConfigurationList and dataProcessingList and some additional parameters that can be re-used or referenced later in the file (referenceableParamGroupList), and (ii) the run element which includes the spectrumList and the chromatogramList (optional) elements (Fig. 2). Each spectrum item defined in the spectrumList has two attributes to access this element, an index and the id, which are mandatory. Both can act as a unique identifier for the spectrum; however, we recommend that developers use the id element as the primary unique identifier for a spectrum, especially when using jmzML (see below), since some file manipulations could alter the index attribute but the id value should never change. The different attributes (e.g. MS level for each scan) are specified in the CV parameters called cvParam. Finally, the raw data is compressed (into Base64 binary encoding) and defined by the binary tags specified in the binaryDataArrayList. To access mzML files, several implementations have been developed by different groups, providing APIs for the parsing of these files such as jmzML. The most common tasks required by developers at this stage are to retrieve spectral data, determine the type of data for each scan (MS^1^ or MS^2^), retrieve the retention time, and for MS^2^ scans retrieve the parent ion *m*/*z*. In Fig. 2, we provide some examples to show where these elements are contained in a typical mzML file, with annotated Java code for simple file processing using jmzML. The extraction of specific spectral data will be discussed in detail in the next section.

### 2.2 Building quantitative software that processes large mzML files

A quantitative experiment usually comprises several technical or biological replicates [39]. Considering an average mzML file of 3 GB in size and six replicates, we will be processing nearly 20 GB in the experiment. Researchers at the European Bioinformatics Institute (EBI) have developed a library called xxindex that is embedded in the jmzML API to improve the speed when accessing each element. This implementation also reduces the memory overhead associated with loading XML files into memory. The library contains a dictionary of XPath (path of tags used to locate an element in the file) entries used to identify all the XML elements with the corresponding byte position (location) in the file at which they reside. In this library, only the locations of the indexes are stored in memory rather than the entire file. For this approach, the loading time is often proportional to the complexity of the XML structure (i.e. complex XML structures will take more time). The process of converting byte-stream data into objects, known as ‘unmarshalling’, can take up to several minutes (~ 3–4 min for a 3 GB mzML file in an Intel® Core™ 2 Quad CPU at 2.83 GHz with 8 GB of RAM). However, the benefit is that future calls to a specific spectrum are instantaneous as the object location is stored in memory and the overall memory overhead is low.

**Fig. 3:**
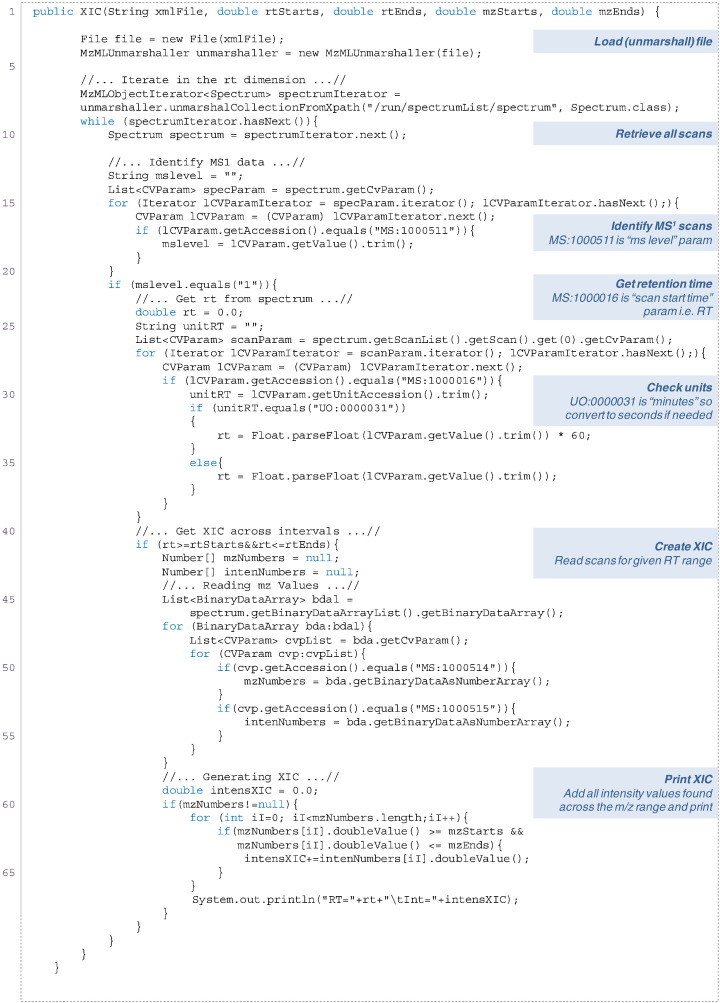
Example code showing how an extracted ion chromatogram (XIC) can be generated from an mzML file, using jmzML. The code takes input parameters of an RT and *m*/*z* range.

So far we have only describe the use of jmzML for reading specific elements, however, sometimes we will need to read the raw data to extract a region in two dimensional space (i.e. *m*/*z* and RT) (Fig. 3). To select a specific region we can specify the parameters of the spectra we wish to retrieve, for example, the first 1000 scans. Then, we can iterate over each specific scan and retrieve the corresponding *m*/*z* and intensity values. Fig. 3 shows how the jmzML can be used to convert binary-encoded data into arrays of doubles and how an extracted ion chromatogram can be generated from a portion of the file, specified by a range of *m*/*z* and RT space.

### 2.3 Accessing identification data in mzIdentML

The mzIdentML standard (stable version 1.1 [26]) has been developed to act as a standard output format generated from peptide and protein identification tools, such as sequence database search engines, and a standard input to post-processing and quantitation software that are reliant on external tools for performing the identifications. The number of software supporting mzIdentML standard is growing (http://www.psidev.info/tools-implementing-mzidentml), which encourages proteome bioinformatics developers to produce tools that support mzIdentML, rather than native support for processing all the individual search engine formats. Native export of mzIdentML version 1.1 is supported by Mascot [40] (in version 2.4 and above), OpenMS [41], PEAKS [42], MSGF + [43], and Scaffold [44] and external converters exist for OMSSA [45] and X!Tandem [46] in the mzidLib project [47] for Phenyx from GeneBio [48], for SEQUEST/ProteomeDiscoverer in ProCon [49] and the Trans-Proteomic Pipeline via ProteoWizard [31]. In this section, we briefly describe the data model for PSMs and grouped protein identifications in mzIdentML, alongside example code snippets for extracting information using jmzIdentML.

**Fig. 4:**
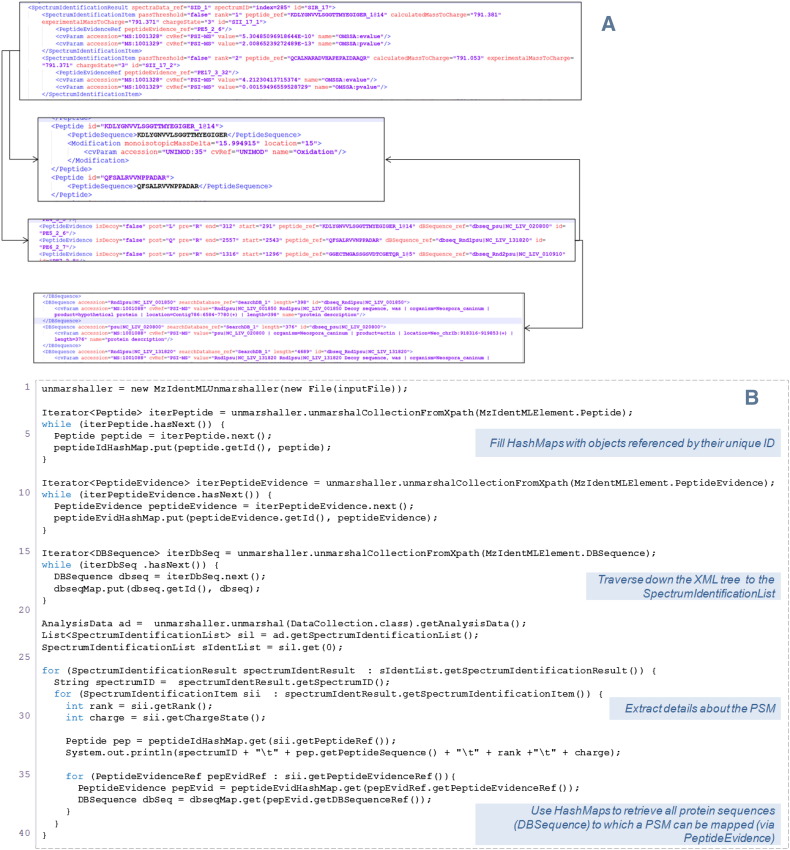
(A) Example of mzIdentML capturing PSMs in SpectrumIdentificationItem (SII). SII has references to the Peptide sequence and PeptideEvidence. PeptideEvidence is a one-to-many mapping from a Peptide sequence to proteins, captured in DBSequence. (B) Code snippets using jmzIdentML to retrieve all PSMs from a file. Note: jmzIdentML has a configuration file, allowing references between objects to be switched on or off (auto-resolving). In the example, all object references have been switched off (lowest memory overhead) — requiring the use of internal HashMaps for retrieving objects by their unique ID.

All peptide-level identifications in mzIdentML are contained within a structure called the SpectrumIdentificationList within which the elements of type SpectrumIdentificationResult reside to capture the peptides identified for each individual spectrum (Fig. 4A). One single PSM is captured in a SpectrumIdentificationItem (SII), which has an attribute rank to show the ordering of all hits reported for the spectrum and a passThreshold attribute used to indicate whether the hit is above or below the threshold (e.g. p < 0.05), specified elsewhere in the file (Fig. 4A). Other attributes contained in the SII include chargeState, calculatedMassToCharge and experimentalMassToCharge. The SII captures the scores associated with the identification, such as e-values or ‘ion scores’, using CV parameters, sourced from the PSI-MS CV. SII uses the peptide_ref attribute to reference the peptide that was identified from this spectrum, stored within a re-usable element called Peptide. SII also references PeptideEvidence elements that capture the mapping from a peptide sequence to the protein sequences, stored in the DBSequence element, in which it can be found.

**Fig. 5:**
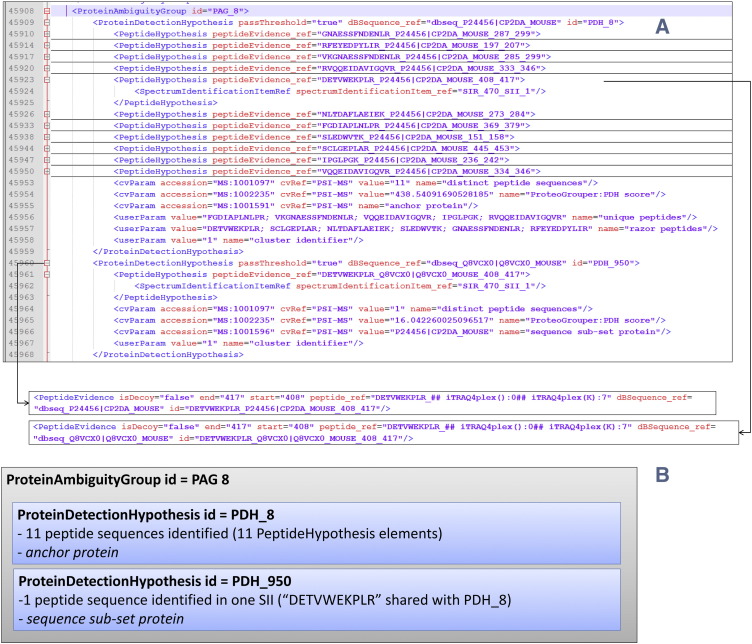
(A) An overview of how protein groups are represented in an mzIdentML, showing two proteins in one group, one of which (PDH_8) has been identified by 11 peptides in many spectra (not all shown) and a second protein (PDH_950) has only been identified by one peptide (DETVWEKPLR) in one spectrum only. This peptide is shared with PDH_8, hence the protein is flagged as “sequence sub-set protein” and PDH_8 as the “anchor protein” for the group. A) Snippet of mzIdentML; B) graphical representation of the groups shown.

Following a protein inference step, results are captured in ProteinDetectionList. All results are stored within elements called ProteinAmbiguityGroup (PAG), where each PAG captures a grouped set of protein identifications within which there is ambiguity in peptide to protein inference (Fig. 5). Grouping protein identifications in this way is required since it is common for a protein to share some or all of the peptide identifications with other proteins. The PAG thus represents the evidence for a single detected isoform, even though several different (putatively identified) database accessions may be contained within the group. Peptides shared between proteins arise from gene families with closely related sequences, the database storing different splice products of the same gene, database errors (e.g. multiple records for the same gene products) and chance matches of typically short peptides in otherwise unrelated proteins.

A single identification of a protein accession from the source database is represented in ProteinDetectionHypothesis (PDH) element, which sits within a PAG in the hierarchy, along with any associated scores or statistical values, captured as CV terms. The PDH references the DBSequence element (not shown), which captures the native accession of the protein in the source database and, optionally, the protein sequence itself, description line and other basic details that can be extracted from the source database (such as a fasta file). Additionally, a CV term “anchor protein” is used for flagging that one PDH is the group representative. CV terms can be used to express relationships for other PDHs to the group representative, such as “sequence same-set”, “spectrum same-set”, “sequence subset” and “spectrum subset”. Following the style of code used in Fig. 4, Java code can be written in a straightforward manner to parse the ProteinDetectionList, and retrieve all PAGs and all contained PDH elements.

### 2.4 Representing quantitative data in mzQuantML

The primary purpose of PSI's mzQuantML is to provide the capability to communicate quantitative values attached to proteins or groups of proteins (that share some ambiguity in peptide assignment), peptides (potentially derived from multiple MS measurements) and quantified regions of raw MS data, called ‘features’. A typical quantitative proteomics report could thus consists of (i) tables of values on proteins, (ii) peptides and (iii) features, each measured across a number of replicates (technical and/or biological), captured in an element called Assay or potentially averaged for each type of independent variable (i.e. a grouping of replicates), captured in an element called StudyVariable. The mzQuantML format has structures to capture all of these data types and connections amongst the quantitation levels. The way data is encoded in mzQuantML has slight differences dependent upon the type of experimental technique employed, including label-free or labelling, MS^1^ based or MS^2^ based. Each of these types of techniques is encoded in a similar manner in mzQuantML, although due to the nature of underlying techniques, each encoding requires some specific guidelines to ensure consistency.

The mzQuantML format has recently passed the final stages of the PSI's standardisation process [50] resulting in version 1.0 release (Feb 2013). The corresponding Java API, jmzQuantML is being developed in tandem, offering similar functionality to the other APIs as described above.

The complexity in working with mzQuantML comes from the variability in different quantitative workflows that need to be encoded. To cope with the variability, the standard is formally defined by several specifications that work in a complementary mode. First, the structure of an XML file is formally governed by an XML Schema, as for all other XML-based PSI standards. Second, CV terms have to be used in specific places in the file — these are defined by ‘mapping files’, formally defining which CV terms must, should or may be used in different parts of the format, according to each type of experimental workflow. Lastly, a set of semantic rules have been defined (in plain text) modulating the structure of the file for (i) label-free intensity workflows, (ii) label-free spectral counting workflows; (iii) MS^1^ labelling techniques, such as SILAC and (iv) MS^2^ tagging approaches, such as iTRAQ [51] or TMT [52]. The semantic rules can be checked by validation software to ensure that each type of mzQuantML file is consistently structured.

**Fig. 6:**
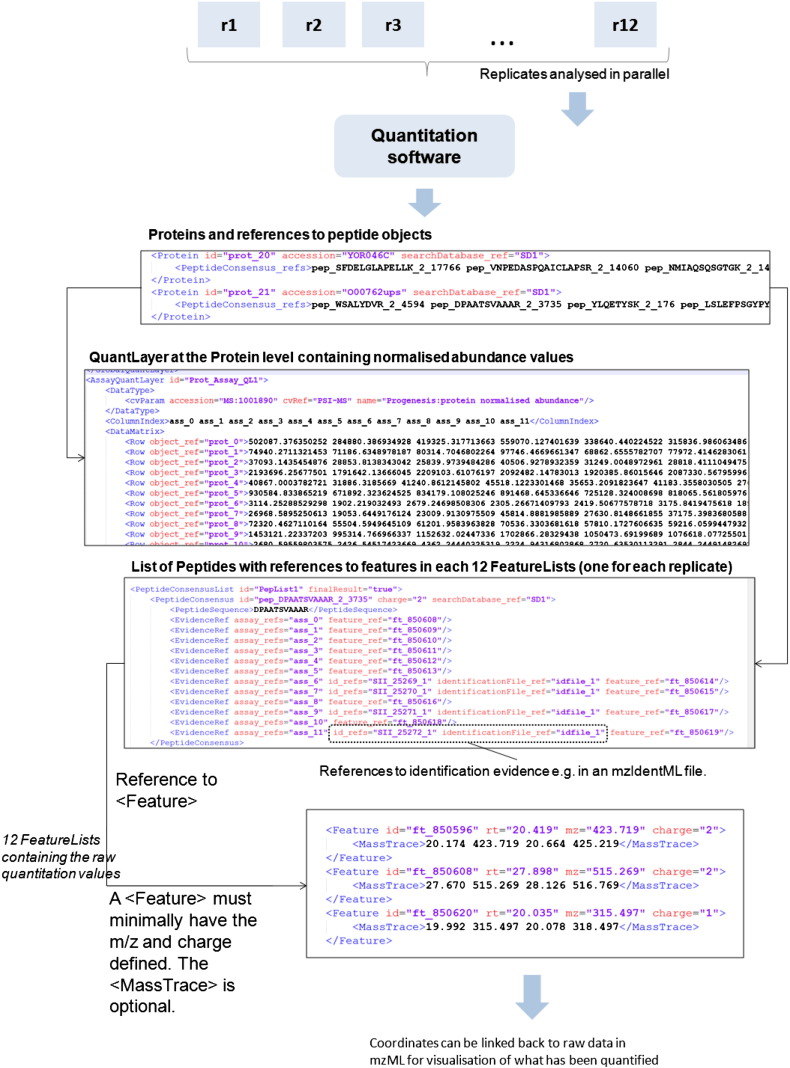
A workflow showing the encoding of quantitative data in mzQuantML for a label-free experiment in which 12 replicates are analysed to produce abundance values at the protein and peptide level (peptide level QuantLayer not shown).

In this section, we describe only how label-free feature-based quantitation workflows can be encoded in mzQuantML, for technical details on other workflows, the project website should be consulted [27]. The core of mzQuantML is the storage of quantitative data in structures called QuantLayers. A file can contain a list of all proteins (or groups of proteins) quantified, a list of peptides quantified or a list of ‘features’ quantified — corresponding to regions in two-dimensional RT-*m*/*z* space. In the most straightforward example, a QuantLayer within the protein (or peptide) lists capture data across all replicates in a data matrix (or table), where the rows of the table are individual proteins (say) and the columns are the replicate measures. As shown in Fig. 6, a QuantLayer typically stores only one data type at a time, in this case normalised abundance values. In this label-free analysis, twelve MS runs were performed (called Assay elements in mzQuantML), thus producing twelve columns in the data matrix. If 1000 proteins have been quantified, the table would have dimensions of 12 columns × 1000 rows, with data values accessible by their X and Y coordinates. If there are multiple protein-level data types, each one is modelled in a new QuantLayer, meaning that the overall structure is consistent. Each Protein element has references to the Peptide elements on which the quantitation was based. The QuantLayer described is specific to Assays, hence called an AssayQuantLayer. Other types of QuantLayer are available for storing ratios (a RatioQuantLayer) or for storing data combined over several assays, called a StudyVariableQuantLayer — for more details see the specification documents and tutorial material [27]. Lastly, a GlobalQuantLayer structure is available for storing multiple data types about proteins (or peptides) that are common across all runs or study variables (or not specific to any one run or study variable), such as p-values.

Data for individual features are stored within a FeatureList that has a one-to-one correspondence with one MS run i.e. in this example, there would be twelve FeatureList elements. A Feature can be described by the *m*/*z* of the monoisotopic peak and the centre point of the RT. The exact coordinates that have been quantified in two-dimensions can be captured in MassTrace, thus enabling the interpretation of how the software has quantified the feature and the development of visualisation software for overlaying results on top of the raw data, for example stored in mzML.

The overall structure of mzQuantML thus can consist of a complete trace back from final values — such as abundance values for each protein grouped across replicates, back to protein abundance values in each run, peptide abundance values (in each run) and regions of raw data (*m*/*z* versus RT space) that have been quantified. This level of detail may not be required for all users, but by capturing this level of detail, it is envisaged that capabilities for comparing, re-analysing and verifying complex quantitative data analyses routines can be improved.

## 3 Discussion

The primary drivers behind the development of PSI standard data formats are to make proteomics data open to (re)analysis by groups other than those who produced the data, to support more general open access to scientific data and to streamline software development so that developers do not have to support an ever growing number of file formats if they want their software to have impact across the field, or if they want to optimise just one part of a full workflow. The three formats reviewed in this article — mzML, mzIdentML and mzQuantML are all represented in XML, they all use CV parameters primarily sourced from the PSI-MS CV and have some complexity of inter-relationships between elements. The references between elements exist to reduce redundancy in the file so that an element representing the same underlying concept does not need to be repeated, thus producing smaller file sizes and easier transfer into software models or databases. However, the downside of the number of relationships between elements means that there is a steeper learning curve for developers to understand the models. Coding software for import/export of the standards without using an API can be challenging. In this article, we have briefly described the most important features of the standards, required for developers working in quantitative software development. For those developers working in Java, the APIs may be used for both file reading and writing, since they have been designed specifically to manage large file sizes. Although only briefly mentioned here, the APIs can be tailored to specific needs through the use of configuration files. For relatively small files, each API can be configured so that references between objects are automatically resolved, allowing the developer to write code for moving seamlessly around the XML tree via references between objects as needed. For larger files, on the order of GBs for raw data, such configuration is not suitable, since this requires loading the full XML tree into memory. The APIs can be thus configured to load only what is needed, retrieving elements from disk based on an index of their position within the file.

For developers working in other programming languages, more tools and resources are constantly being released. The ProteoWizard project effectively provides a C++ API for manipulating raw data and can write output into mzML as needed [31]. ProteoWizard also exports from a number of existing identification formats into mzIdentML, and it is anticipated that ProteoWizard will support mzQuantML when this format is formally released as a version 1.0. The OpenMS project also supports mzML, mzIdentML and mzQuantML, providing various tools for reading and writing these formats [41]. For developers working outside of C++ or Java, there are currently fewer tools available for working with the standards, although these are starting to emerge, such as a Python library for mzML [53]. The PSI groups with responsibility for each of the standards are also aware of the need for access to the standards from programmers working in non-object oriented languages. While most programming languages have general XML libraries available, there is a clearly considerable detail in the standards, which developers would have to learn. To support these developers through an alternative route, tools are under development providing interfaces for converting to and from comma-separated values (CSV) or tab-separated files, which also provide a suitable route in statistical packages, such as ProteoWizard which contains a text-based converter to mzML (txt2mzml) and the mzidLib project which provides converters to and from CSV files for mzIdentML under active development (http://code.google.com/p/mzidentml-lib/).

## 4 Conclusions

The PSI is developing guidelines and standard formats to assist data sharing and open-source development in proteomics. This article reviews the most important concepts from three formats, expected to be used by developers working on entire quantitation pipelines or modules within larger projects. The mzML and mzIdentML formats are both stable and starting to become well supported across the proteomics community. The mzQuantML format has been recently finalised in early 2013 to support re-analysis and post-processing of quantitative data and capturing a full trace of the manipulations performed on data. These formats are all somewhat (unavoidably) complex, since proteomics is a complex science with no concept of a ‘typical’ experimental or analysis pipeline. As more developers work with the PSI standards for software development, we envisage a critical mass of software emerging around the standards, making data sharing, re-analysis of data and open-source development considerably more straightforward. The PSI is an open organisation, and anyone with an interest in contributing directly to the development of standards or tools for working with the standards is encouraged to join the relevant mailing list (see www.psidev.info) or attend one of the PSI annual meetings.

## Figures and Tables

**Table 1:** Availability of Java APIs for HUPO-PSI standards described in the article.

Tool	Version	Description	URL	Publication
jmzML	1.1	Java API for manipulating mzML files	http://code.google.com/p/jmzml/	[32]
jmzIdentML	1.1	Java API for manipulating mzIdentML files	http://code.google.com/p/jmzidentml/	[34]
jmzQuantML	1.0.0	Java API for manipulating mzQuantML files	http://code.google.com/p/jmzquantml/	N/A^a^

a jmzQuantML is released at version 1.0.0 status but not yet published.
